# Supplementary material for: Adaptation to genome decay in the structure of the smallest eukaryotic ribosome
Source: Nat Commun. 2022 Feb 1;13:591. doi: 10.1038/s41467-022-28281-0 (PMC8807834; doi:10.1038/s41467-022-28281-0)
Supplement: Supplementary file 1 — Supplementary Information [file 41467_2022_28281_MOESM1_ESM.pdf]

Supplementary Information

Adaptation to genome decay
in the structure of the smallest eukaryotic ribosomes

Authors
David Nicholson[1], Marco Salamina[2], Johan Panek[2], Karla Helena-Bueno[2], Charlotte R. Brown[2], Robert P. Hirt[2], Neil A. Ranson[1], Sergey V. Melnikov[2,3]

Affiliations
[1] Astbury Centre for Structural Molecular Biology, School of Molecular & Cellular Biology, Faculty of Biological Sciences, University of Leeds, Leeds LS2 9JT, UK
[2] Biosciences Institute, Newcastle University, Newcastle upon Tyne, NE2 4HH, UK
[3] Faculty of Medical Sciences, Newcastle University, Newcastle upon Tyne, NE2 4HH, UK

# To whom correspondence should be addressed:
n.a.ranson@leeds.ac.uk
sergey.melnikov@ncl.ac.uk

Key words:
ribosome ǀ rRNA ǀ microsporidia ǀ genome decay ǀ intracellular parasites

Supplementary figures

Supplementary Figure 1 | Collection and processing of cryo-EM data.

Supplementary Figure 2 | Overall and local resolution assessment of the *E. cuniculi* ribosome maps.

Supplementary Figure 3 | Map vs model curves.

Supplementary Figure 4 | Additional truncations in rRNA and protein segments in *E. cuniculi* ribosomes compared to *Nosematedae* ribosomes from *P. locustae* and *V. necatrix*.

Supplementary Figure 5 | Progressive degeneration of a ribosomal protein upon transition from free-living fungi with large genomes to microsporidians with extremely reduced genomes.

Supplementary Figure 6 | Local changes in ribosomal protein structure compensate for rRNA reduction.

Supplementary Figure 7 | Summary of evolutionary changes in the structure of *E. cuniculi* ribosomes.

Supplementary Figure 8 | A spermidine-like small molecule at the protein-protein interface in the interior of the *E. cuniculi* ribosome.

Supplementary tables

Supplementary Table 1 | Validation of the *E. cuniculi* ribosome structure.

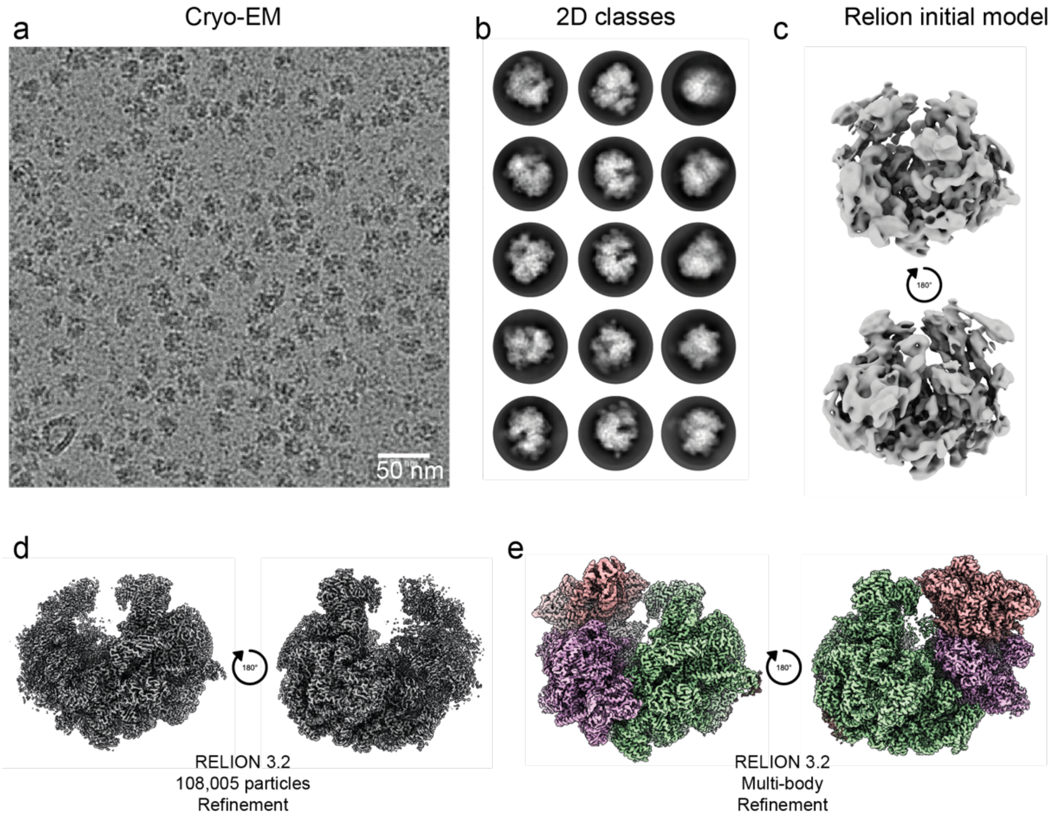

Supplementary Figure 1 | Collection and processing of cryo-EM data. (**a**) Representative cryo-electron microscopy micrograph of *E. cuniculi* ribosomes. This micrograph represents one of the 2,210 micrographs comprising the experimental dataset (**Methods**). (**b**) Representative cryo-EM 2D class averages (**c**) and initial model generated in RELION 3.1. Cryo-EM density following (**d**) 3D refinement and (**e**) multi-body refinement. Final refinement was performed on 108,005 particles extracted from 2,210 micrographs in RELION 3.1. The 3 bodies "LSU", "SSU-head" and "SSU-body" masks were used for multibody refinement.

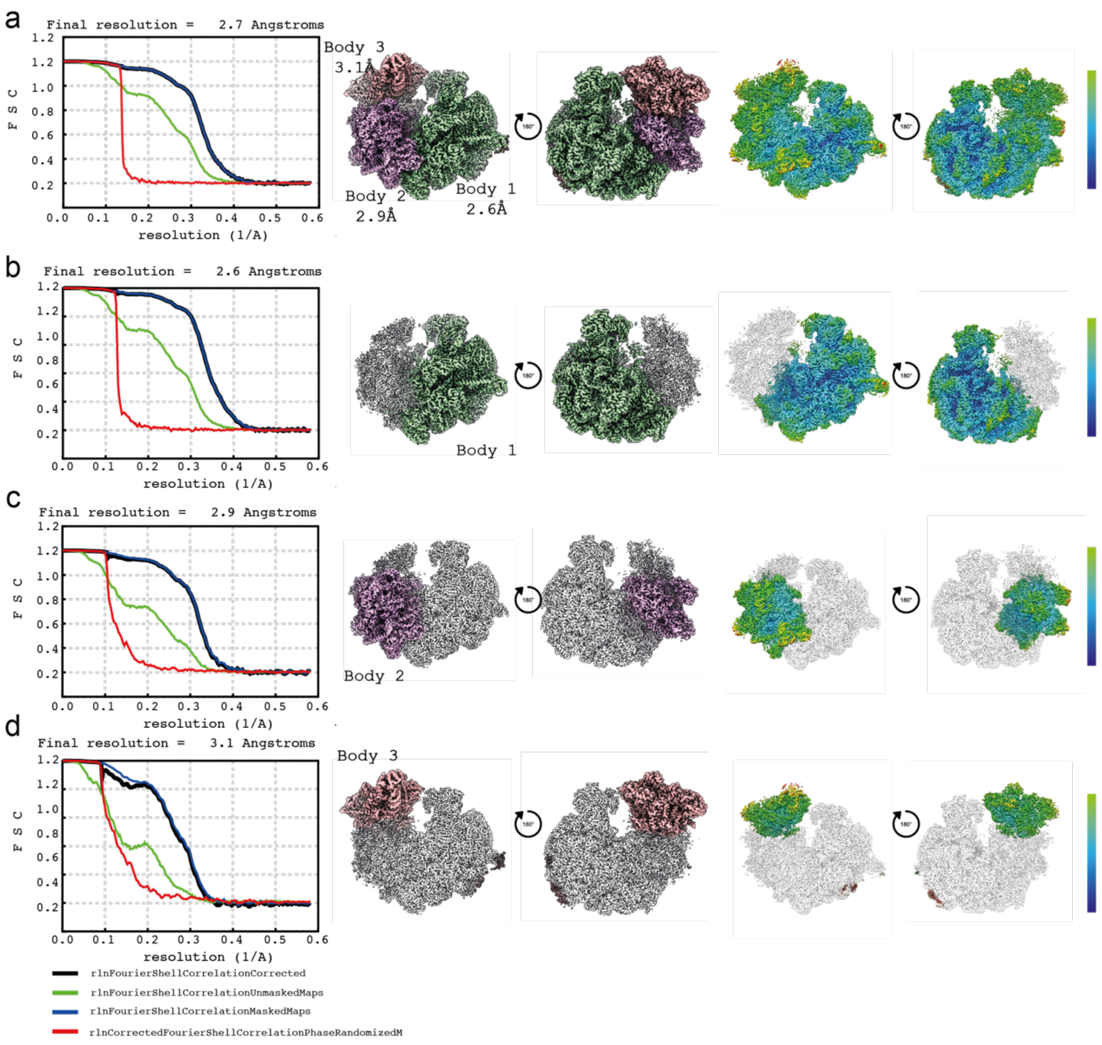

Supplementary Figure 2 | Overall and local resolution assessment of the *E. cuniculi* ribosome maps.
(**a-d**) Overall and local resolution estimations of (**a**) the consensus map, (**b**) the LSU map, (c) the SSU-body map, (**d**) the SSU-head map. The panel on the left shows Fourier Shell Correlation (FSC) curves for the respective map. An overview of the cryo-EM map is shown next to a slab view and two 180-degree related views colored according to local resolution. Local resolution was calculated using RELION 3.1. The bar indicates resolution, ranging from 2.3Å (dark blue) to 2.9Å (light green).

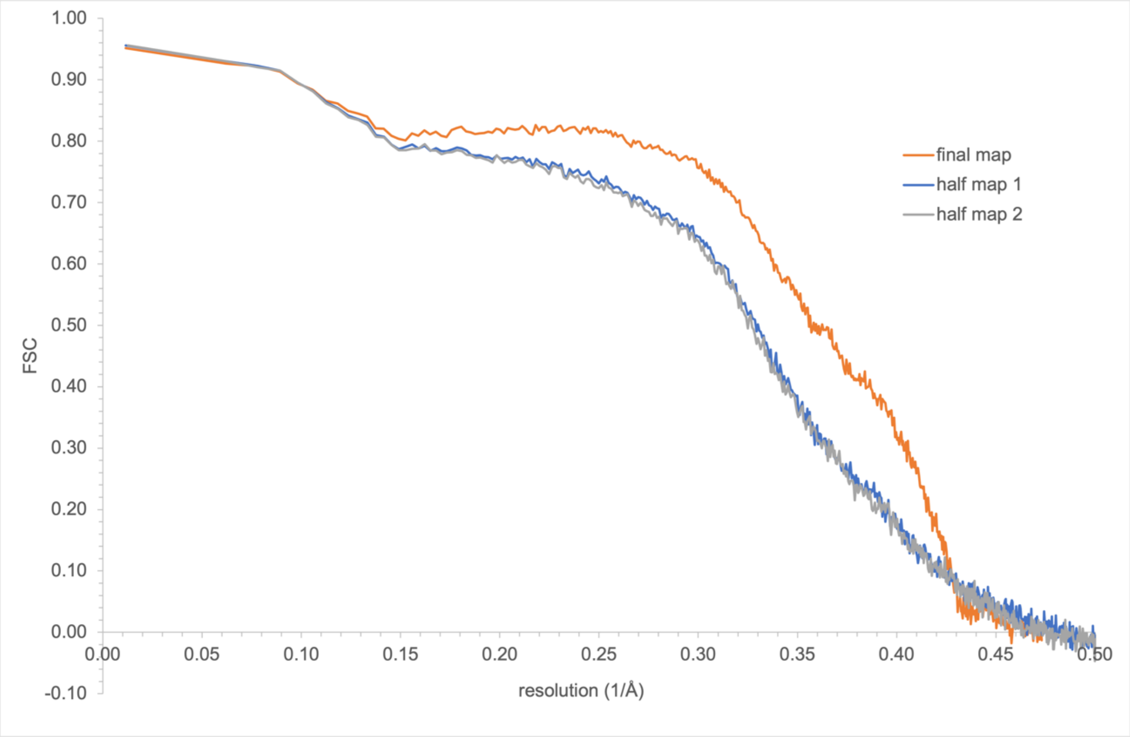

Supplementary Figure 3 | Maps vs model curves. FSC vs 1/Å resolution curves for the full consensus map (i.e. local resolution filtered map), and the two half maps against the final atomic model of *E. cuniculi* ribosomes. The 'model resolution' is defined as the FSC(0.5) map vs model value, corresponding to 2.8Å for main map, and 3.1Å for half maps.

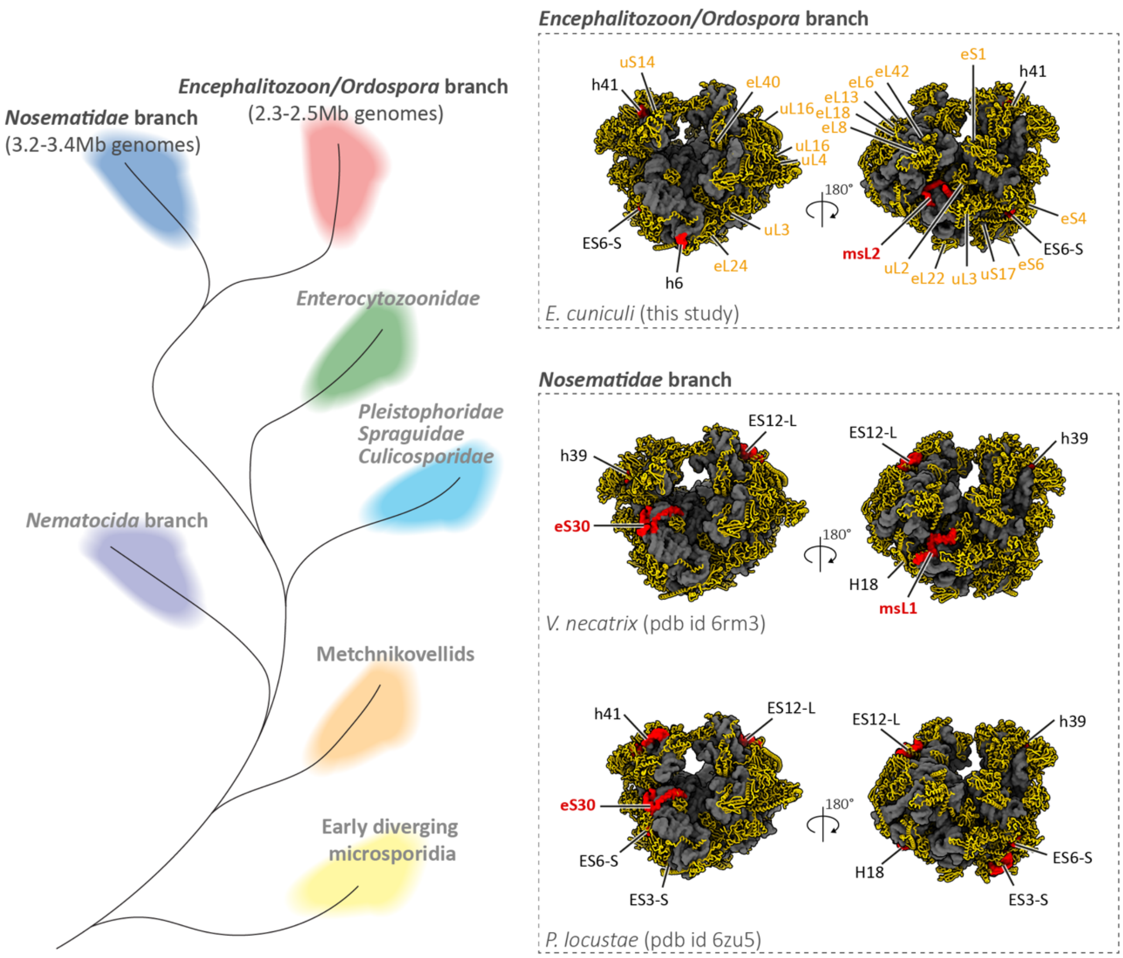

Supplementary Figure 4 | *E. cuniculi* ribosomes possess additional truncations in rRNA and protein segments compared to *Nosematedae* ribosomes from *P. locustae* and *V. necatrix*. Microsporidian tree of life is shown to illustrate *Encephalitozoon/Ordospora* branch of microsporidian species, which are known for their smallest and most reduced genomes among eukaryotic species.

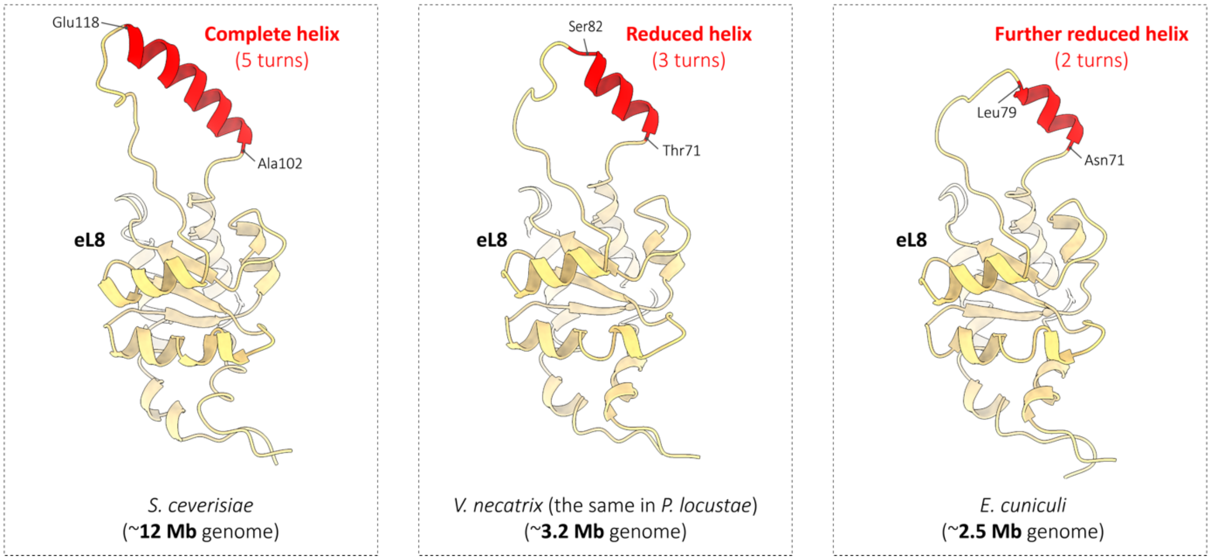

Supplementary Figure 5 | Progressive degeneration of a ribosomal protein upon transition from free-living fungi with large genomes to microsporidians with extremely reduced genomes.

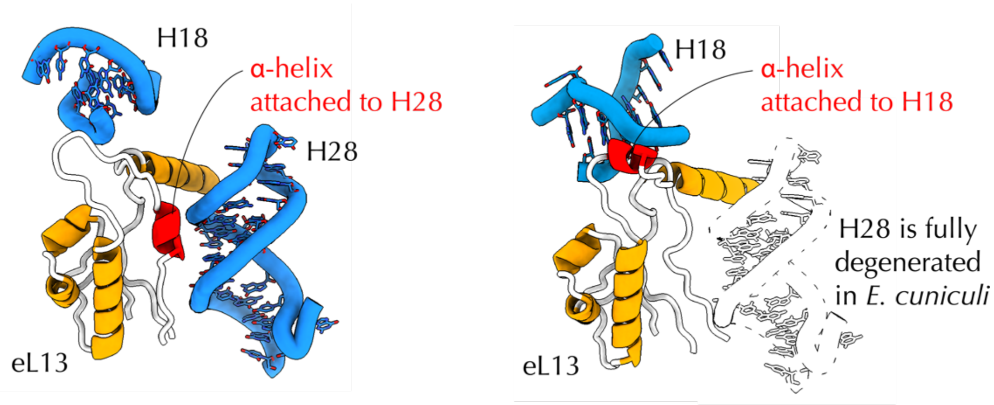

Representative eukaryote
(*S. cerevisiae*)

Microsporidia
(*E. cuniculi*)

Supplementary Figure 6 | Microsporidian ribosomal proteins reinvent their rRNA attachment sites as an apparent adaption to rRNA degeneration.

Previously, we described a mechanism by which ribosomes adapt to the evolution of new rRNA segments in eukaryotic ribosomes. We showed that eukaryotic proteins (compared to their bacterial homologs) remodeled their solvent-exposed loops into miniature α-helices. These helices serve as new binding sites for eukaryote-specific rRNA expansions, ensuring a proper folding of rRNA expansions in the ribosome.

Here, we found that the same strategy is used by *E. cuniculi* ribosomes to help adapt ribosomes to rRNA degeneration. For example, protein eL13 converts one of its loops into an α-helix that binds the truncated rRNA. This structural change allows eL13 to reinforce its binding to the *E. cuniculi* ribosome in which the primary binding site of eL13 is eliminated due to the loss the rRNA expansion $ES7^L$ (and the helix H28).

This finding shows that, by transforming their structure back and forth between the states of "loop" and "α-helix", ribosomal proteins can create and eliminate rRNA binding sites, thus heling rRNA to expand or shrink without loss of the ribosome integrity. This strategy may explain numerous changes in ribosomal proteins from other species that possess anomalously long or short rRNA molecules.

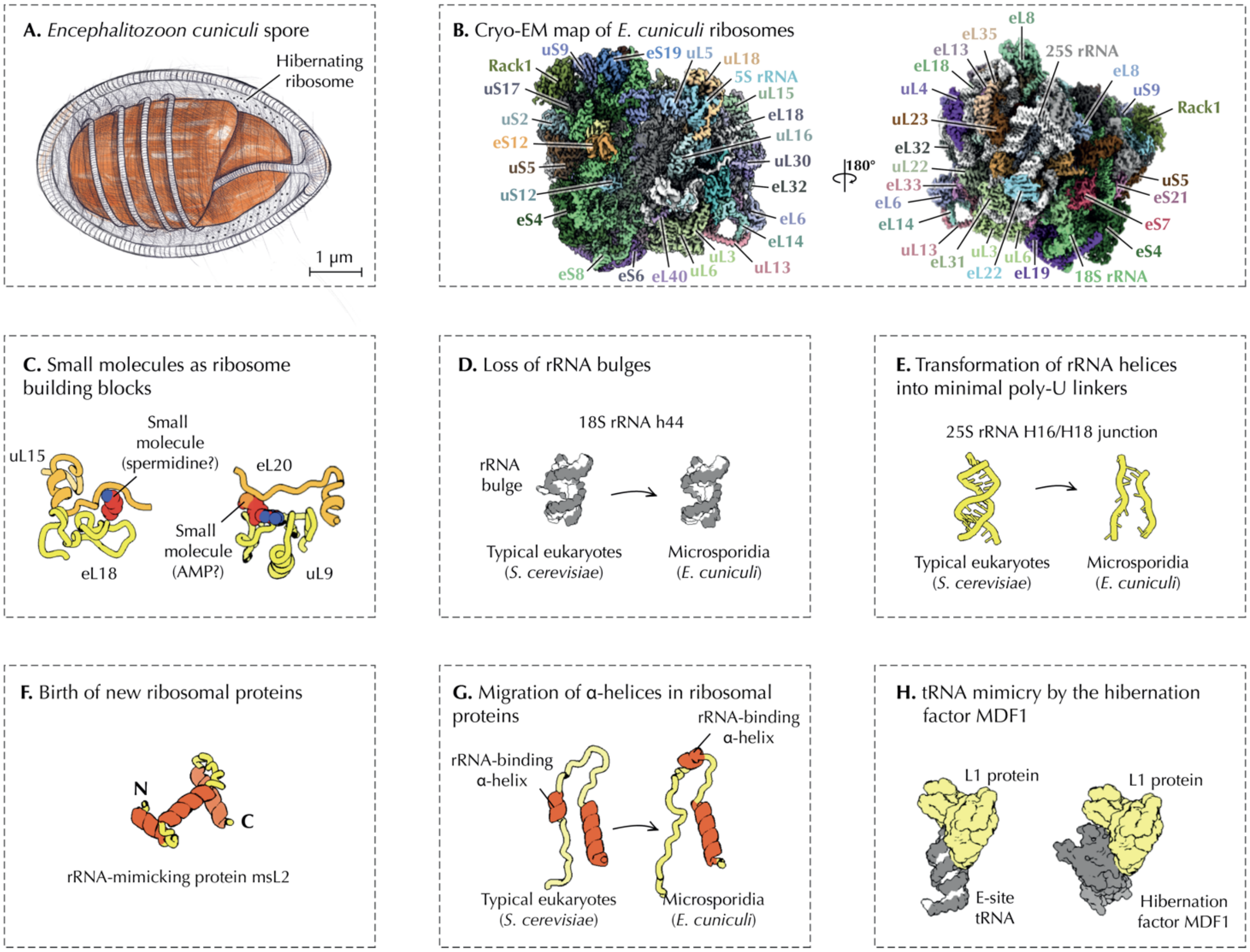

Supplementary Figure 7 | Electron microscopy reveals major evolutionary innovations in the miniaturized ribosomes from the human pathogen *Encephalitozoon cuniculi*. (**A**) Schematic structure of an infectious spore of the human pathogen *E. cuniculi.* In this study, *E. cuniculi* spores were used to purify ribosomes bound with the hibernation factor Mdf1. (**B**) A cryo-EM map of *E. cuniculi* ribosomes in complex with the hibernation factor Mdf1. (**C**-**G**) The map reveals previously unknown structural features of microsporidian ribosomes and improves our understanding of how nucleic acids and proteins adapt to or suffer from extreme genome reduction in intracellular forms of life. (**H**) The map also reveals that the hibernation factor Mdf1 mimics tRNA molecules upon binding to the ribosome.

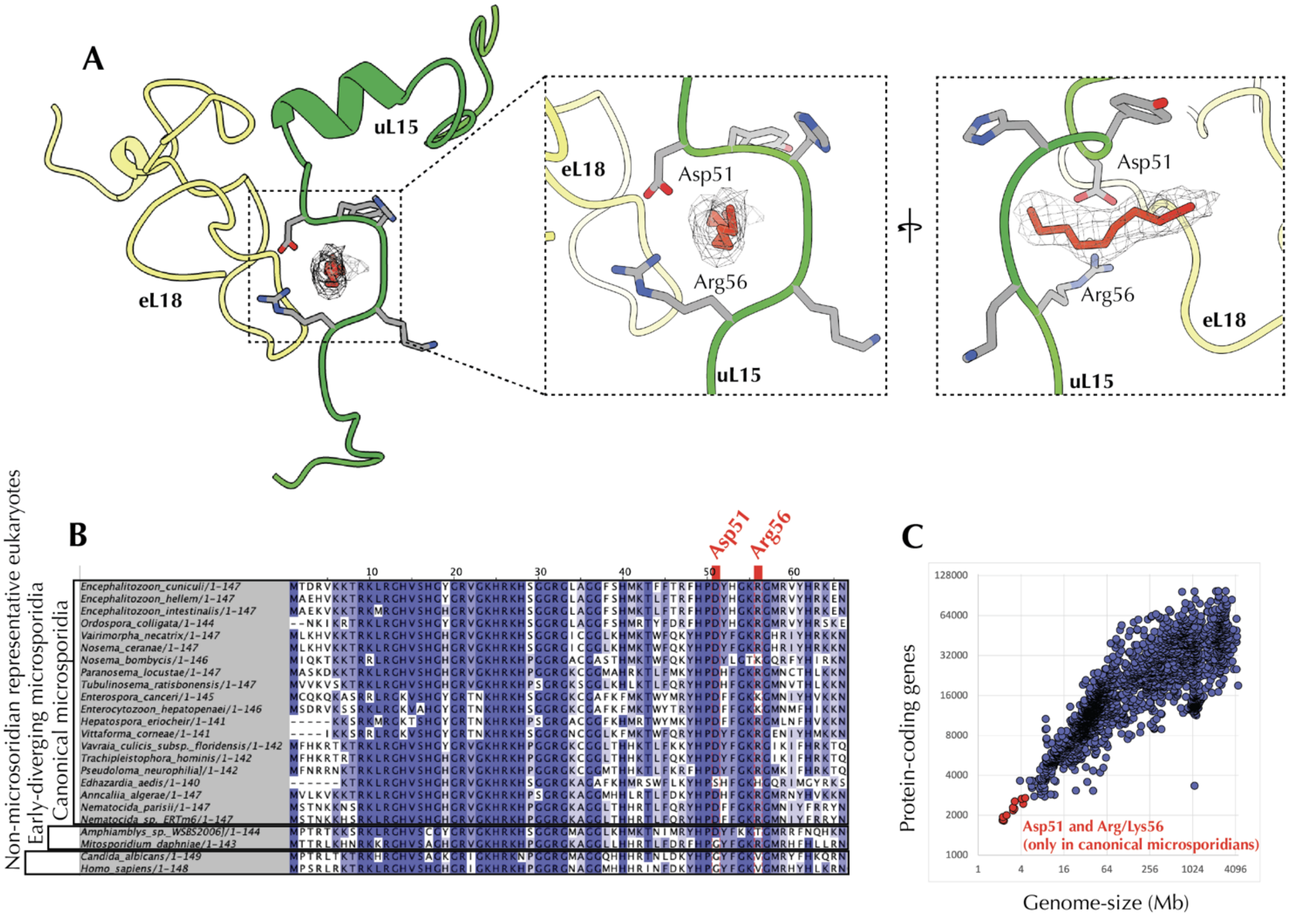

Supplementary Figure 8 | A spermidine-like small molecule trapped at a protein-protein interface in the interior of the *E. cuniculi* ribosome. (**A**) A fragment of the cryo-EM structure shows the cryo-EM map (contoured at 0.1V) of a small molecule that is trapped in the ribosome interior between ribosomal proteins uL15 and eL18. A salt-bridge between the residues Asp51 and Arg56 in uL15 creates a donut-like binding site for this molecule in the ribosome structure. In other eukaryotic ribosomes (e.g. in yeasts and humans), this void is occupied by the C-terminal extension of protein eL18, which is truncated in *E. cuniculi*. (**B**) A multiple sequence alignment shows high conservation of Asp51 and Arg56 residues in microsporidian species: of all microsporidian homologs of uL15 (which we could identify through protein-protein NCBI blast search), these residues are conserved in nearly all microsporidian species, aside from *Edhazardia aedis* and early diverging microsporidians. In other microsporidian species, Asp51 and Arg56 residues are either immutable or show Arg56 mutation to a similar residue, Lys56. (**C**) The plot schematically shows eukaryotic species plotted according to their genome size. The panel illustrates that Asp51 and Arg56/Lys56 residues are observed only in microsporidian species with highly reduced genomes (red dots) and not in other species (blue dots).

	E. cuniculi ribosome (EMDB-13936) (PDB 7QEP)
**Data collection and processing**	
Magnification	96,000
Voltage (kV)	300
Electron exposure (e–/Å^2)	60
Defocus range (μm)	-0.8 to -2.6
Pixel size (Å)	0.861
Symmetry imposed	C1
Initial particle images (no.)	278,672
Final particle images (no.)	108,005
Map resolution (Å)	2.7
FSC threshold	0.143
Map resolution range (Å)	5.4 to 2.3
**Refinement**	
Initial model used (PDB code)	4V88 & 6RM3
Model resolution (Å)	2.8
FSC threshold	0.5
Model resolution range (Å)	Infinity to 2.8
Map sharpening *B* factor (Å^2)	-80.0
Model composition	
Non-hydrogen atoms	166129
Protein residues	10885
Ligands	spermidine, AMP
B factors (Å^2)	
Protein	19.6
Ligand	18.7
R.m.s. deviations	
Bond lengths (Å)	0.009
Bond angles (°)	0.943
Validation	
MolProbity score	2.00
Clashscore	8.35
Poor rotamers (%)	0.09
Ramachandran plot	
Favored (%)	90.10
Allowed (%)	9.69
Disallowed (%)	0.21

Supplementary Table 1 | Cryo-EM data collection, refinement and validation statistics.
