# Supplementary material for: Adaptation to genome decay in the structure of the smallest eukaryotic ribosome
Source: Nat Commun. 2022 Feb 1;13:591. doi: 10.1038/s41467-022-28281-0 (PMC8807834; doi:10.1038/s41467-022-28281-0)
Supplement: Supplementary file 3 — Description of Additional Supplementary Files [file 41467_2022_28281_MOESM3_ESM.pdf]

Description of Additional Supplementary Files

File Name: Supplementary Data 1
Description: A script to retrieve sequences of ribosomal proteins from UniProt.

File Name: Supplementary Data 2
Description: Multiple sequence alignments of eukaryotic eL20 sequences.

File Name: Supplementary Data 3
Description: Multiple sequence alignments of eukaryotic uL15 sequences.
