# Supplementary material for: Adaptation to genome decay in the structure of the smallest eukaryotic ribosome
Source: Nat Commun. 2022 Feb 1;13:591. doi: 10.1038/s41467-022-28281-0 (PMC8807834; doi:10.1038/s41467-022-28281-0)
Supplement: Supplementary file 7 — Reporting Summary [file 41467_2022_28281_MOESM7_ESM.pdf]

nature portfolio

Corresponding author(s): Sergey Melnikov

Last updated by author(s): Jan 7, 2022

Reporting Summary

Nature Portfolio wishes to improve the reproducibility of the work that we publish. This form provides structure for consistency and transparency in reporting. For further information on Nature Portfolio policies, see our Editorial Policies and the Editorial Policy Checklist.

Statistics

For all statistical analyses, confirm that the following items are present in the figure legend, table legend, main text, or Methods section.

n/a	Confirmed	
☐	☒	The exact sample size (*n*) for each experimental group/condition, given as a discrete number and unit of measurement
☒	☐	A statement on whether measurements were taken from distinct samples or whether the same sample was measured repeatedly
☒	☐	The statistical test(s) used AND whether they are one- or two-sided<br>*Only common tests should be described solely by name; describe more complex techniques in the Methods section.*
☒	☐	A description of all covariates tested
☒	☐	A description of any assumptions or corrections, such as tests of normality and adjustment for multiple comparisons
☒	☐	A full description of the statistical parameters including central tendency (e.g. means) or other basic estimates (e.g. regression coefficient) AND variation (e.g. standard deviation) or associated estimates of uncertainty (e.g. confidence intervals)
☒	☐	For null hypothesis testing, the test statistic (e.g. *F*, *t*, *r*) with confidence intervals, effect sizes, degrees of freedom and *P* value noted<br>*Give P values as exact values whenever suitable.*
☒	☐	For Bayesian analysis, information on the choice of priors and Markov chain Monte Carlo settings
☒	☐	For hierarchical and complex designs, identification of the appropriate level for tests and full reporting of outcomes
☒	☐	Estimates of effect sizes (e.g. Cohen's *d*, Pearson's *r*), indicating how they were calculated

Our web collection on statistics for biologists contains articles on many of the points above.

Software and code

Policy information about availability of computer code

Data collection	The sequences of ribosomal proteins were collected using a custom python script deposited in Supplementary Data 1.
Data analysis	The sequences of ribosomal proteins were aligned using Clustal Omega 1.2.2 (https://www.ebi.ac.uk/Tools/msa/clustalo/)<br>The cryo-EM snapshots were processed using Rellion 3.1 (https://www3.mrc-lmb.cam.ac.uk/relion/index.php/Main_Page), CTFFIND4 (https://grigorieffla b.umassmed.edu/ctffind4), and DeepEMhancer (using Tensorflow 1.14) (https://gith ub.com/rsa nchezgarc/deepEM hancer)<br>The atomic structure of E. cuniculi ribosomes was built using Coot 0.8.9.1 (https://www2.mrc-lmb.cam.ac.uk/personal/pemsley/coot/), and and analyzed using ChimeraX 1.2.5 (https://www.cgl.ucsf.edu/chimerax/), and MolProbity 4.5.1 (http://molprobity.biochem.duke.edu/).

For manuscripts utilizing custom algorithms or software that are central to the research but not yet described in published literature, software must be made available to editors and reviewers. We strongly encourage code deposition in a community repository (e.g. GitHub). See the Nature Portfolio guidelines for submitting code & software for further information.

Data

Policy information about availability of data

All manuscripts must include a data availability statement. This statement should provide the following information, where applicable:

- Accession codes, unique identifiers, or web links for publicly available datasets
- A description of any restrictions on data availability
- For clinical datasets or third party data, please ensure that the statement adheres to our policy

Data that were used in this study include PDB ID 4v88 for the structure of S. cerevisiae ribosomes and PDB ID 6rm3 for the structure of V. necatrix ribosomes. Data that support the finding of this study have been deposited in the EMDB/PDB databank with the accession code PDB ID 7QEP for the structure of the E. cuniculi ribosome, and EMD-13936 for the corresponding cryo-EM maps. The authors claim no conflict of interest.

Field-specific reporting

Please select the one below that is the best fit for your research. If you are not sure, read the appropriate sections before making your selection.

- [x] Life sciences
- [ ] Behavioural & social sciences
- [ ] Ecological, evolutionary & environmental sciences

For a reference copy of the document with all sections, see nature.com/documents/nr-reporting-summary-flat.pdf

Life sciences study design

All studies must disclose on these points even when the disclosure is negative.

Sample size	In this study, we used 2,210 cryo-EM micrographs of E. cuniculi ribosomes, with the total of 108,005 particles used to calculate cryo-EM maps.
Data exclusions	The initial dataset containing 278,672 particles was reduced to 108,005 particles using masks of the large and the small ribosomal subunits for Laplacian-of-Gaussian autopicking.
Replication	Cryo-EM structures are determined as an average of a large number of particles (108,005 particles in this study) and do not require replication.
Randomization	The randomization is not applicable to this study because our experimental design did not include techniques and approaches that require randomization.
Blinding	The blinding is not applicable to this study because our experimental design did not include techniques and approaches that require blinding.

Reporting for specific materials, systems and methods

We require information from authors about some types of materials, experimental systems and methods used in many studies. Here, indicate whether each material, system or method listed is relevant to your study. If you are not sure if a list item applies to your research, read the appropriate section before selecting a response.

Materials & experimental systems

n/a	Involved in the study
x	Antibodies
	x Eukaryotic cell lines
x	Palaeontology and archaeology
x	Animals and other organisms
x	Human research participants
x	Clinical data
x	Dual use research of concern

Methods

n/a	Involved in the study
x	ChIP-seq
x	Flow cytometry
x	MRI-based neuroimaging

Eukaryotic cell lines

Policy information about cell lines

Cell line source(s)	The cell line of rabbit kidney cells RK13 was acquired from the American Type Culture Collection (accession number CCL-37™)
Authentication	The cell line used in this study was not authenticated.
Mycoplasma contamination	The cell line was cultivated in the presence of antibacterial antibiotics and was not tested for Mycoplasma contamination.
Commonly misidentified lines (See ICLAC register)	N/A
